# Quinone-fused porphyrins as contrast agents for photoacoustic imaging†
†Electronic supplementary information (ESI) available. See DOI: 10.1039/c7sc01369h



**DOI:** 10.1039/c7sc01369h

**Published:** 2017-06-27

**Authors:** Srinivas Banala, Stanley Fokong, Christian Brand, Chrysafis Andreou, Bernhard Kräutler, Magnus Rueping, Fabian Kiessling

**Affiliations:** a Institute for Experimental Molecular Imaging , University Clinic , RWTH Aachen University , Pauwelstraße 30 , D-52074 Aachen , Germany . Email: sbanala@ukaachen.de ; Fax: +49 241 803380116 ; Tel: +49 241 8085566; b Institute of Organic Chemistry , RWTH Aachen University , Landoltweg 1 , D-52074 Aachen , Germany; c Department of Radiology , Memorial Sloan Kettering Cancer Center , 1275 York Avenue , New York , NY 10065 , USA; d Institute of Organic Chemistry , University of Innsbruck , Innrain 80-82 , A6020 , Innsbruck , Austria; e KAUST Catalysis Center (KCC) , King Abdullah University of Science and Technology (KAUST) , Thuwal 23955-6900 , Saudi Arabia

## Abstract

Photoacoustic (PA) imaging is an emerging non-invasive diagnostic modality with many potential clinical applications in oncology, rheumatology and the cardiovascular field. For this purpose, there is a high demand for exogenous contrast agents with high absorption coefficients in the optical window for tissue imaging, *i.e.* the near infrared (NIR) range between 680 and 950 nm. We herein report the photoacoustic properties of quinone-fused porphyrins inserted with different transition metals as new highly promising candidates. These dyes exhibit intense NIR absorption, a lack of fluorescence emission, and PA sensitivity in concentrations below 3 nmol mL^–1^. In this context, the highest PA signal was obtained with a Zn(ii) inserted dye. Furthermore, this dye was stable in blood serum and free thiol solution and exhibited negligible cell toxicity. Additionally, the Zn(ii) probe could be detected with an up to 3.2 fold higher PA intensity compared to the clinically most commonly used PA agent, ICG. Thus, further exploration of the ‘quinone-fusing’ approach to other chromophores may be an efficient way to generate highly potent PA agents that do not fluoresce and shift their absorption into the NIR range.

## Abstract 2

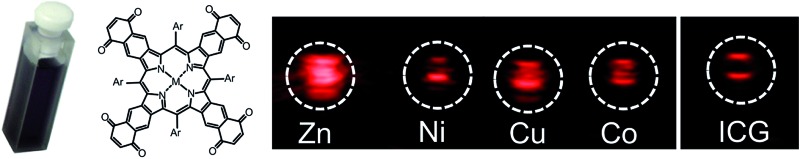
Naphthoquinone fused porphyrins showed higher photoacoustic signals than ‘standard’ indocyanine green (ICG). In this context, the insertion of Zn(ii) resulted in the most potent photoacoustic dye, which also proved to be biocompatible and stable in serum.

## Introduction

Photoacoustic imaging (PAI) is a non-invasive biomedical imaging technology,^1^ which benefits from the high sensitivity of optical imaging and the excellent tissue penetration of ultrasound (US).^2,3^ In PAI, a light absorbing material, either of endogenous or exogenous origin, is irradiated by short (nanosecond) low energy laser pulses, leading to the transfer of the absorbed energy to the surrounding tissue in a non-radiative process causing a small rise (a few mK) in temperature. This temperature rise generates thermo-elastic expansion and acoustic pressure waves which are collected using standard US transducers and processed as PA images.^4^ Clinical trials are ongoing with the endogenous contrast of haemoglobin to assess tissue vascularization and to detect tissue hypoxia.^5^ Exogenous contrast agents expand the application field of PAI, *e.g.* to sentinel lymph node detection and the molecular characterization of tumors.^6,7^ In this context, strongly NIR-absorbing pigments like polydopamine have been suggested.^8^

**Fig. 1:**
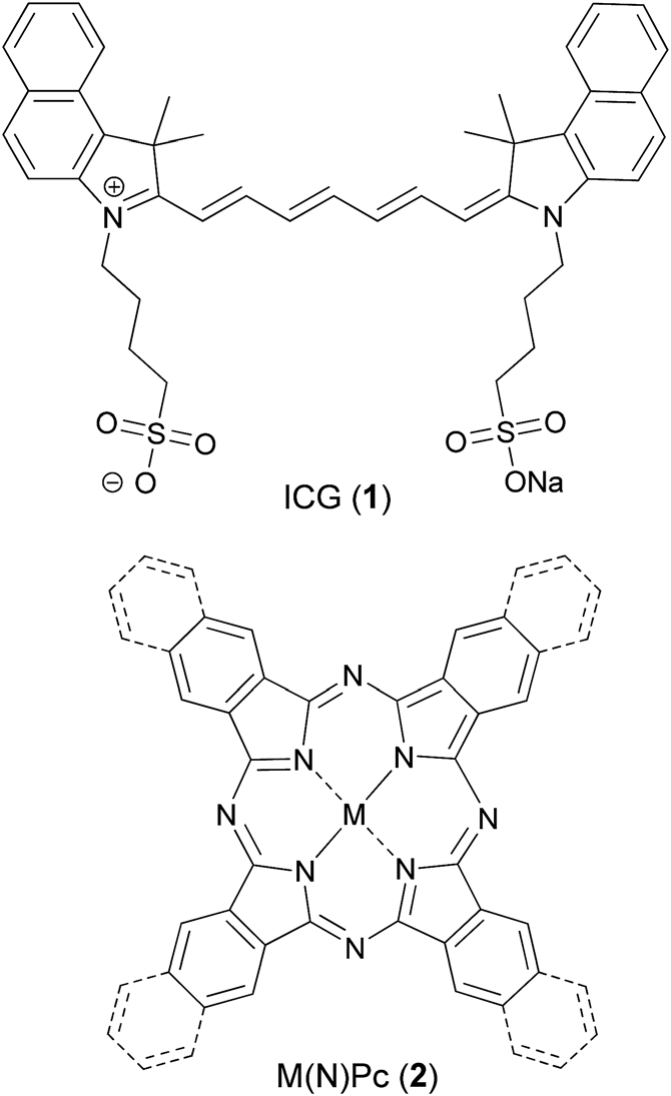
Structures of studied ‘organic’ PA chromophores.

Alternative coloured pigments are tetrapyrroles, which act as key ligands in many biologically active complexes, such as oxygen binding heme, light harvesting photosystems in chlorophyll and redox reactions in cytochromes. Thus, this class has been intensely studied for bioinorganic,^9^ medicinal^10^ and material applications.^11^ Porphyrins show strong absorption, in particular for visible wavelengths, and fluorescence emission in the red to near infrared (NIR) range. Peripheral modification may generate panchromatic pigments, which are interesting for a variety of applications.^12,13^ As a basis for the synthesis of NIR chromophores, porphyrins are envisaged with a variety of linkages,^13^ in which an anthraquinone as a bridging synthon can be applied.^14^ Tetra naphthoquinone-conjugated porphyrins absorb in the whole visible and NIR range,^15,16^ thus, their appearance in organic solutions (CH_2_Cl_2_/methanol) is black. Therefore, and due to the absence of fluorescence emission, the application of such dyes for biomedical photoacoustic imaging is highly promising (Fig. 1).

Within the PAI field, intense research is dedicated to the development of new probes. To date, mostly metallic nanoparticles of gold, silver, copper and other heavy metals have been evaluated.^17^ However, unfavourable pharmacokinetic and pharmacodynamic properties as well as toxicological concerns often limit their translation to clinical applications.^18^ Organic dyes can overcome these limitations. Indocyanine green (ICG, **1**) has been widely studied for PAI, owing to its FDA approval for clinical use in fluorescence microscopy and angiography.^19^ Nevertheless, the short blood half-life (*t*_1/2_ < 3 min), nonspecific binding to serum proteins and the higher non-PA contributing fluorescence emission make ICG a non-ideal chromophore for PAI. Although the clinical translation of PAI is already ongoing, several physical aspects of PA signal generation of probes are still not fully understood. Typically, low fluorescence quantum yields with high molar absorption coefficients and sharp NIR-absorption peaks are preferred. Due to their interesting absorption, polymethine-based IR dyes and proprietary fluorophores (ATTO dyes, Alexa Fluors), which were previously designed for fluorescence imaging applications, have been studied for PAI application. Hence, their success in PAI may be limited.^20^ Similarly, black hole quencher (BHQ) dyes have also been explored.^21^ Thus, most of the work regarding organic chromophores is based on a trial and error approach.

Previously, NIR-absorbing highly fluorescent squaraine-based dyes^22–24^ and (aza)BODIPY dyes were studied for PA generation.^25–27^ However, their fluorescence emission reduces PA signal generation. Similarly, we and others have evaluated phthalocyanines (Pcs)^28,29^ and naphthalocyanines (NPcs, **2**) which exhibit intense NIR-absorption^30^ but generate singlet oxygen (^1^O_2_) and other reactive oxygen species (ROS) upon irradiation.^10^ This ROS generation is useful for photodynamic therapy but adversely affects their suitability for diagnostic PA applications. Additionally, core-modified NIR-absorbing porphyrins, *e.g.* quinoline-annulated porphyrins and morpholinobacteriochlorins, have been investigated,^31^ but they also produce ROS. In another study, a chlorin-based pigment was conjugated with a phospholipid to self-assemble into microbubbles, and their US and PA properties towards theranostic applications were studied.^32^ Organic nanoparticles based on photovoltaic materials have attracted attention for PAI due to their strong absorption in the NIR-range.^33^

We conceived probes based on quinone-fused porphyrins to quench fluorescence and simultaneously avoid ROS activity. This modification shifted the absorption of the novel chromophore into the NIR-range, making it suitable for PAI applications. In this manuscript, we report our studies on quinone-fused porphyrins as highly suitable chromophores for PAI for the first time.

## Results and discussion

The quinone-fused porphyrins (**3-M**) are novel panchromatic organic chromophores, which exhibit strong absorption in the NIR-range with negligible fluorescence (for **3-Zn**, see Fig. S4†).^15,16^ Porphyrins typically show changes in their photophysical properties with complexed metals in the core, as seen for black porphyrins **3-2H** and **3-M** (M = Ni and Zn). As the quinone moiety exhibits electron accepting and excited state quenching properties, the dyes do not show ^1^O_2_/ROS generation upon excitation with light, which is highly beneficial for purely diagnostic applications. Therefore, we set out to study the PA efficiency of **3-2H** and **3-M** as a function of different metals (M = Co, Cu, Ni and Zn) and compare them to ICG.

### Synthesis and optical properties

**Scheme 1:**
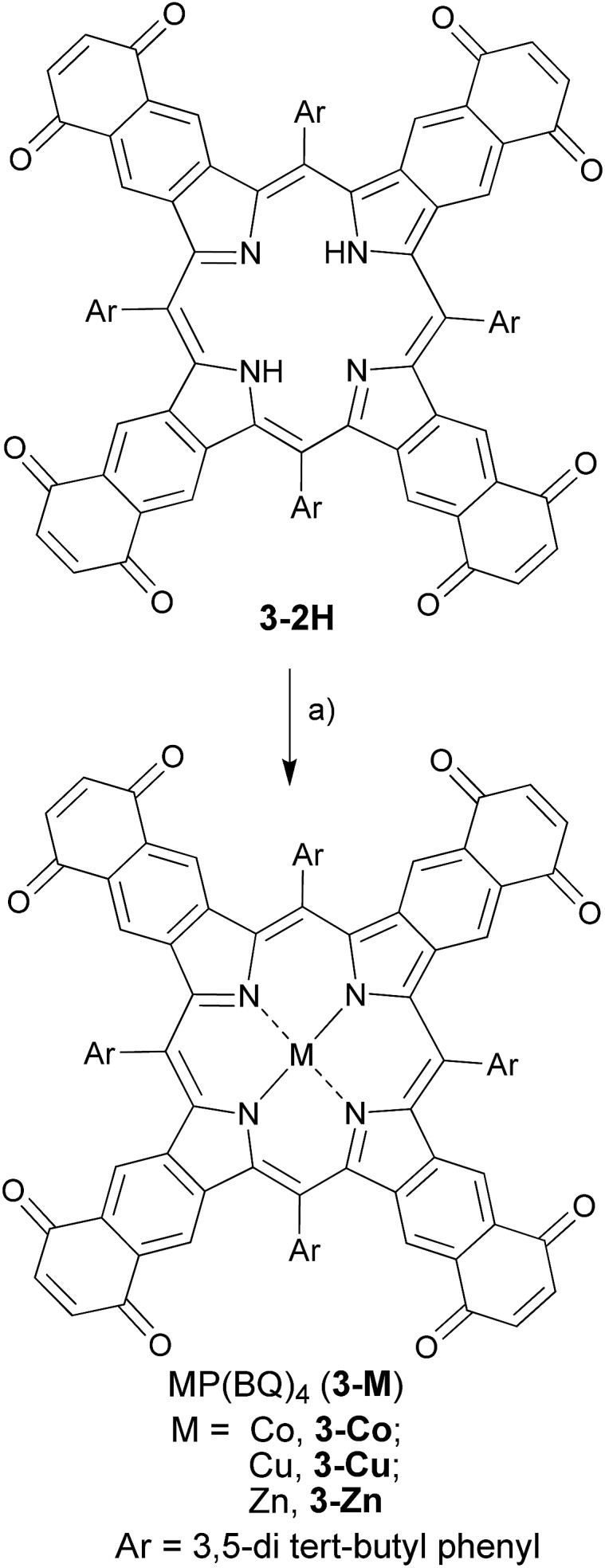
Synthetic route towards ‘black’ metalloporphyrins: (a) M(OAc)_2_ in CH_2_Cl_2_/MeOH/reflux for M = Zn, Cu; CoBr_2_ in THF/NEt_3_/reflux for M = Co.

**Fig. 2:**
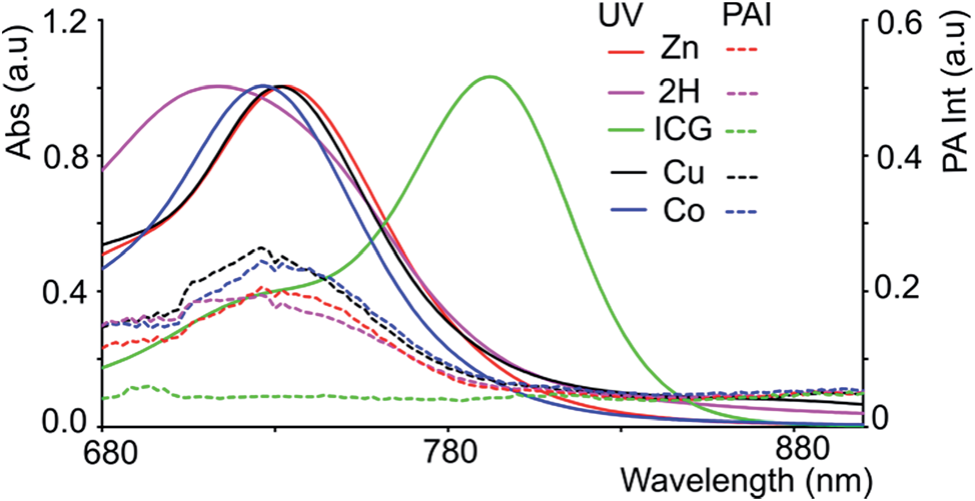
NIR part of the absorption spectra (bold lines) of the ‘black’ porphyrins **3-M** and ICG at *λ*_max_ with O.D. = 1 (in DMF in a 1 cm cuvette, conc. (in μM) of ICG = 10.6, **3-2H** = 30.6, **3-Zn** = 20.3, **3-Cu** = 23.3 and **3-Co** = 36) and tube phantom PA spectra (dotted lines) of respective solutions.

The chromophores, metal-free **3-2H** and metallo **3-Ni** were prepared from tetrasulfoleno porphyrin,^16^ which in turn was obtained from a multi-step reaction from glycine.^34,35^ The metal-free quinone porphyrin (**3-2H**) was inserted with metals (M = Cu and Co) by refluxing with Cu(OAc)_2_ in CH_2_Cl_2_/MeOH, and CoBr_2_ in THF/NEt_3_ to prepare **3-Cu** and **3-Co**, respectively (see ESI†) (Scheme 1). The newly obtained chromophores (**3-Cu** and **3-Co**) were purified, precipitated in MeOH/H_2_O and characterized by mass spectrometry as well as UV/vis absorption (see Fig. S1 and S2 in the ESI†). The absorption patterns of **3-M** inserted with all of the tested metals showed the Q-type band maxima in the NIR-region, from 710 to 735 nm (in DMF, see Fig. 2, and for full spectra see Fig. S3†), with molar extinction coefficients as high as 65 000 M^–1^ cm^–1^. The exo-conjugation effect on the absorption properties,^36^ especially the effect of fusing quinones, is subject to further investigation.^37^

### Photoacoustic measurements

**Fig. 3:**
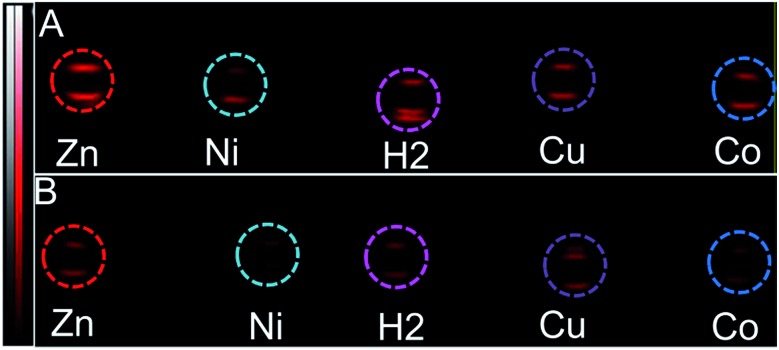
PA images (snapshot at 730 nm) of the tube phantoms containing **3-M**, dilution series (to the conc. in Table 1) (A) 20× dilution in DMF (*i.e.* conc. (in μM) of **3-2H** = 3.2, **3-Zn** = 4.6, **3-Ni** = 6.2, **3-Cu** = 5.3 and **3-Co** = 5.8), (B) 40× dilution in DMF (*i.e.* conc. (in μM) of **3-2H** = 1.6, **3-Zn** = 2.3, **3-Ni** = 3.1, **3-Cu** = 2.7 and **3-Co** = 2.9).

Since black porphyrins show absorption maxima in the PA-suitable NIR region (706–733 nm, see Table 1), although at about 60–80 nm lower than that of ICG, we postulated that the **3-M** series could be good candidates. The molar extinction coefficients (*ε*) of the **3-M** dyes are almost one third of that of ICG, however the absence of fluorescence in **3-M** is expected to compensate for the lower *ε*. To measure their PA properties, we first prepared standard solutions of **3-M** and ICG in DMF (Table 1), as well as equal optical density (O.D. = 1) solutions at their respective NIR wavelength maxima (*i.e.* Abs. at *λ*_max_ = 1 in a 1 cm cuvette, final concentrations range from 10.6 μM to 36 μM). Subsequently, we acquired the PA spectra from 680 to 970 nm using 0.31 mm (i.d.) polypropylene tubes in a water chamber phantom (see ESI Fig. S5†). The PA spectra (Fig. 2) show negligible PA intensity for ICG (0.04 a.u.), which is close to that for the background, whereas for **3-M** it is greater than 0.2 a.u. in the equal O.D. solutions. Next, we measured the PA spectra of the concentrated solutions, normalized the observed PA intensity (at *λ*_max_) by the concentration and divided them with the same ratio of ICG to obtain the relative PA efficiencies for **3-M**. The results are presented in Table 1 and Fig. 3 and S7 in the ESI.† The ‘black’ dyes showed superior PA properties for all dilutions at their absorption maxima. Regarding the metal effect on the PA properties, we found that the Zn(ii) inserted dye generated the strongest signal, providing a 3.2 times higher intensity than that for ICG. This is followed by the metal-free (2.7 times higher), and Cu(ii) (2.4 times higher) containing black dyes. Then, we measured the PA spectra in higher dilutions in order to assess the detection threshold (20 times, *i.e.* 3.2 to 6.2 μM, and 40 times, *i.e.*,1.6 to 3.1 μM). The **3-M** dyes could be detected down to 3 nmol mL^–1^ (see Fig. 3A and B) and **3-Zn** down to 2.3 nmol mL^–1^. For ICG, it was reported that at 10 nmol mL^–1^ was undetectable,^38^ which is in line with our findings (see Fig. 2).

**Table 1:** Optical and photoacoustic properties of **3-M** and ICG

Compd no.	Conc. (μM)	*λ*_max_ [log(*ε*)]^*a*^	At *λ*_max_ (PA Avr)^*b*^	Rel. PA^*c*^
ICG	181	791 (4.97)	2.438	1
**3-Co**	115	733 (4.44)	2.332	1.4
**3-Cu**	106	727 (4.64)	3.449	2.4
**3-Ni**	124	710 (4.37)	2.877	1.7
**3-Zn**	91	732 (4.73)	3.916	3.2
**3-2H**	63	706 (4.51)	2.344	2.7

^*a*^Calculated from the UV/vis measurements of the diluted solutions (4 times for **3-M** and 16 times for ICG) in DMF.

^*b*^The observed PA Avr in a tube phantom (0.31 mm i.d.).

^*c*^Found by normalizing the PA Avr values with the concentration and dividing all of the values with the normalized PA Avr/conc. value for ICG.

**Fig. 4:**
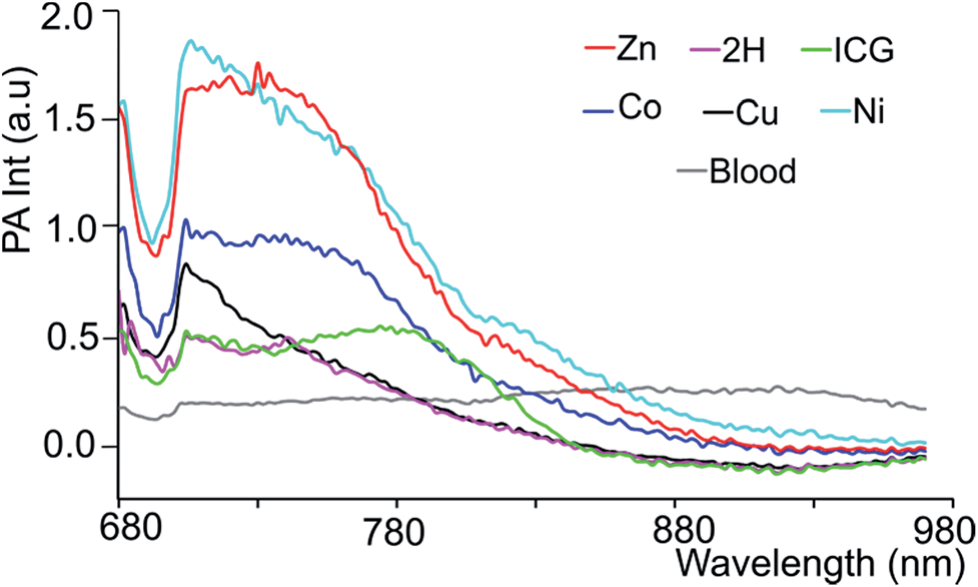
Blood subtracted PA spectra of **3-M** and ICG in blood in a 2 : 1 blood dilution, *i.e.* conc. (in μM) of **3-2H** = 42, **3-Co** = 76.6, **3-Cu** = 70.6, **3-Ni** = 82.6, **3-Zn** = 60.6 and ICG = 120.6.

In order to mimic a natural background, we then studied the PA signals of **3-M** in full swine blood dilutions and compared them to those in DMF dilutions. At 2 : 1 dye-to-blood ratios (40 to 80 μM), up to 5 times higher PA signals than that for ICG (conc. normalized) were obtained for both **3-Ni** and **3-Zn** at their absorption maxima (see Fig. 4). At dye-to-blood ratios of 1 : 4, (15 to 30 μM) PA signals were significantly lower (see Fig. S10†). In comparison to the DMF dilutions, the observed PA signals in blood were about 2.5-fold lower (*i.e.* 15 μM in blood is similar to 6 μM in DMF). This may be due to aggregation of the dyes in blood as well as their adsorption to blood proteins. Moreover, thick and turbid blood suspensions, as was observed in the phantoms, may decrease the transmission and fluence of light, both of which reduce the PA signal intensity.

**Fig. 5:**
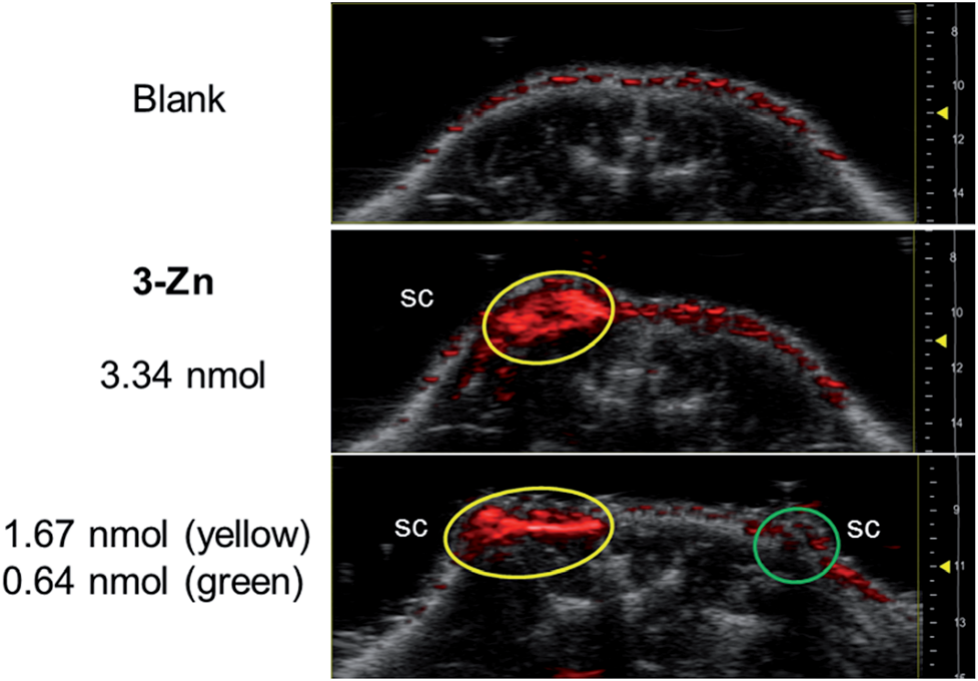
PAI signals (snapshot at 720 nm) of **3-Zn** in dead mice. Subcutaneous (s.c.) injection of **3-Zn** solutions in DMF (for all of the measurements the injection volume = 50 μL). The top blank panel is from before the injection.

Next, PAI was performed on a chicken muscle phantom *ex vivo* after injecting 50 μL of (*ca.* 5 nmol) the **3-M** dye solutions at 2 mm below the surface and adding another layer of chicken muscle on top to increase the depths of the dye deposits. Light transmission and ultrasound attenuation are different in chicken muscle than in water-chamber tube phantoms, thus this can vary the PA signal intensity. In our experiment, the PA signals generated by the **3-M** dyes were strongly visible at a 9–11 mm laser focus depth (see Fig. S12†), where the device delivers intense light, confirming the efficiency of black porphyrins as PAI agents. The PA signals of the black dyes were visible at a depth of 13 mm, where light fluence is low, and vanished at >15 mm, which is due to the focused laser beam design of the device and the unavailability of light. The most sensitive compound, **3-Zn**, was additionally tested in a dead mouse (see Fig. 5 and S13†). 50 μL of differently concentrated solutions were injected subcutaneously (s.c.) or intramuscularly (i.m.) followed by PA imaging. It was found that 1.67 nmol could be detected after s.c. injection (at 1 mm depth) and 3.34 nmol after i.m. injection (at 3 mm depth). The detection limit of **3-Zn** was 0.64 nmol after s.c. injection.

### Biocompatibility

**Fig. 6:**
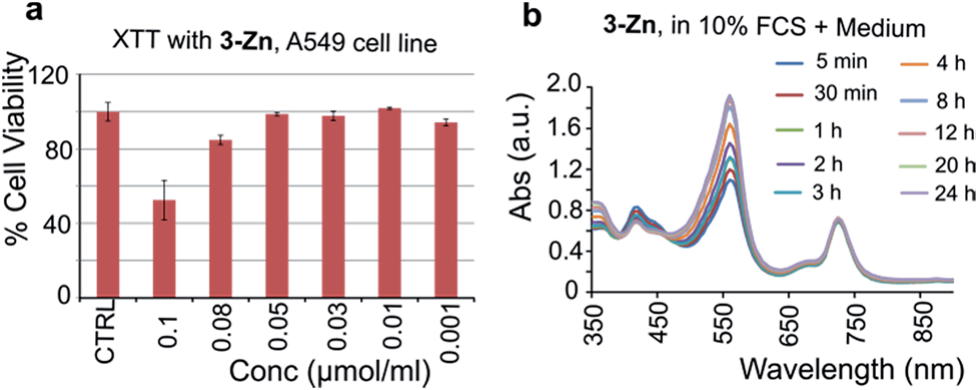
(a) XTT-cell toxicity assay with **3-Zn** in cremophor EL and (b) stability of **3-Zn** in DMEM containing 10% FCS (UV/vis of **3-Zn**).

To test the biocompatibility of **3-Zn**, an XTT (2,3-bis-(2-methoxy-4-nitro-5-sulfophenyl)-2*H*-tetrazolium-5-carboxanilide) cell viability assay was performed using A549 cancer cells after incubation with various concentrations of **3-Zn**. For this purpose, **3-Zn** was incorporated in cremophor EL (2 mg μmol^–1^, **3-Zn**:CrEL) and suspended in phosphate buffered saline (PBS, 1 mM). Almost 100% cell survival was found for up to 0.05 μmol mL^–1^ concentrations (see Fig. 6a), which is far higher than the amount needed for PA generation. Furthermore, the stability of **3-Zn**:CrEL was studied in 10% fetal calf serum (FCS) in DMEM and in PBS using UV/vis spectroscopy (see Fig. 6b, S14 and S15†). No change in the Q band of the absorption spectrum was found for up to 24 h after incubation, indicating the stability of **3-Zn** in a biological environment. Similarly, pristine **3-Zn** was treated with excess free thiol (*N*-acetyl l-cysteine-methylester) in ethyl acetate and was followed by performing supercritical fluid chromatography (SFC, see Fig. S16 and S17†). The SFC chromatogram showed only one peak corresponding to **3-Zn** for up to 8.5 h.

### *In vivo* measurement

**Fig. 7:**
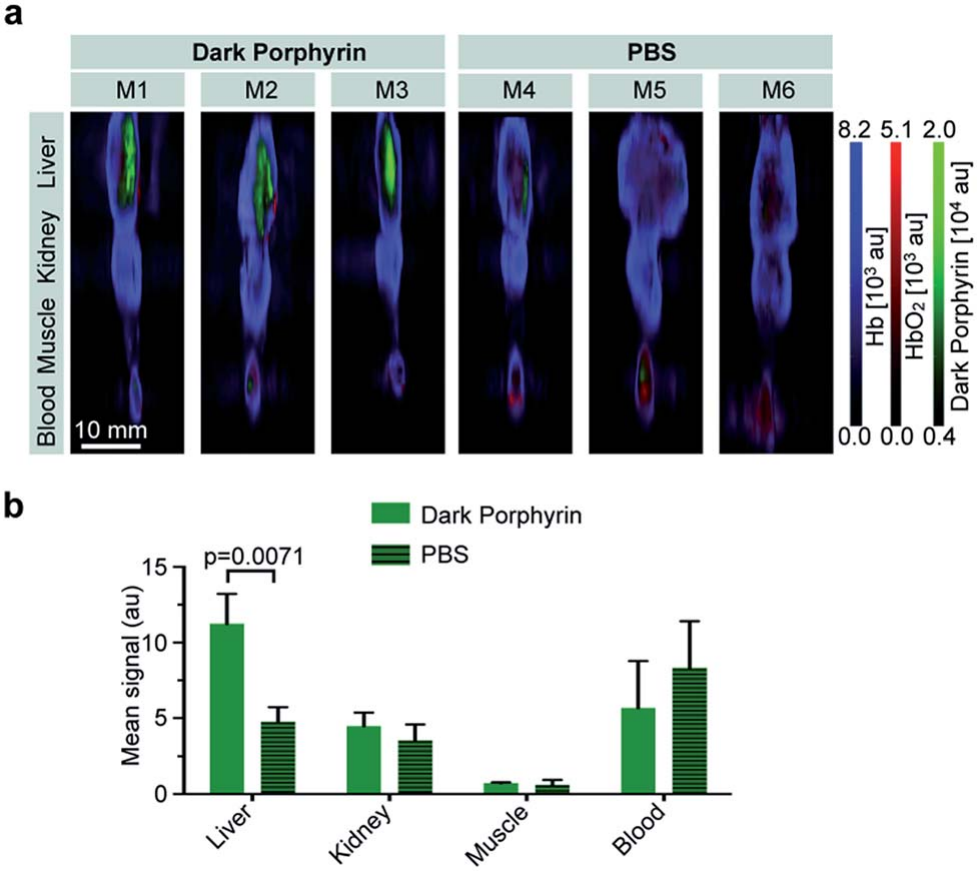
*Ex vivo* MSOT imaging of selected organs. (a) MSOT imaging of liver, kidney, muscle and blood of mice at 1 h post intravenous injection of dark porphyrin **3-Zn** (150 μL, 100 μM, *n* = 3) or PBS (*n* = 3); MSOT images were unmixed for deoxygenated hemoglobin (blue), oxygenated hemoglobin (red) and dark porphyrin (green). (b) Bar graph that compares the detected mean optoacoustic signal of **3-Zn** (unmixed) between mice injected with **3-Zn** and the control (PBS).

Encouraged by these results, we tested **3-Zn** *in vivo*. The quinone-fused dye **3-Zn** (100 μM, 150 μL PBS with 30% PEG300) was intravenously injected into nude mice (*n* = 3). Control mice were injected with PBS (150 μL, *n* = 3) (all of the experiments and procedures were carried out in accordance with the guidelines set by the Institutional Animal Care and Use committee at the Memorial Sloan Kettering Cancer Center, approval number: 13-06-005). At 1 h post injection, the mice were euthanized and blood and selected organs (liver, kidney and muscle) were harvested for *ex vivo* multispectral optoacoustic tomography measurements (MSOT follows the same principle as PA). By linear spectral unmixing, the specific PA signals of **3-Zn**, as well as of oxygenated and deoxygenated hemoglobin, could be detected. The mean intensity values obtained by MSOT were scaled to the same threshold (a.u.) for comparison (Fig. 7a and b). The **3-Zn** injected mice showed a strong signal in the liver, which was not detectable in animals that were injected with PBS alone, indicating that *in vivo* imaging can successfully be performed with this probe. No signals were found in blood, kidney or muscle after 1 h, which can be attributed to the fast liver uptake of this hydrophobic compound. Detailed pharmacokinetic studies are interesting, which is a subject of further investigation. Additional studies with targeting moieties attached to the dye will be carried out.

## Conclusions

We demonstrate for the first time that quinone-fused porphyrins exceed the benchmark probe ICG in providing higher PA signals and being detectable down to 2.3 nmol mL^–1^ in tube phantoms. Of all of the tested quinone-fused porphyrins, the PA performance of **3-Zn** was superior, particularly in terms of its detectability at low concentrations in phantoms, high biocompatibility and serum stability. The versatile synthetic approach used for the construction of this new type of porphyrin allows the introduction of further functional groups and the efficient fine-tuning of the chromophore and, for molecularly-targeted conjugates, specific *in vivo* applications. It is also interesting to consider our quinone-fusing approach to shift the absorption maxima of other chromophores to longer wavelengths (>800 nm), which will lead to better light penetration and imaging deeper structures.
