# Supplementary material for: Quinone-fused porphyrins as contrast agents for photoacoustic imaging
Source: Chem Sci. 2017 Jun 27;8(9):6176–81. doi: 10.1039/c7sc01369h (PMC5628350; doi:10.1039/c7sc01369h)
Supplement: Supplementary file 1 [file SC-008-C7SC01369H-s001.pdf]

Electronic Supplementary Material (ESI) for Chemical Science.
This journal is © The Royal Society of Chemistry 2017

Supporting Information for

Quinone-Fused Porphyrins as Contrast Agents for Photoacoustic Imaging

Srinivas Banala,*[a,b] Stanley Fokong,[a] Christian Brand,[c] Chrysafis Andreou,[c] Bernhard Kräutler,[d] Magnus Rueping,[b] and Fabian Kiessling[a]

[a] *Experimental Molecular Imaging, University Clinic, RWTH Aachen University, D-52074 Aachen, Germany. E-mail: sbanala@ukaachen.de*
[b] *Institute of Organic Chemistry, RWTH Aachen University, Landoltweg 1,Germany.*
[c] *Department of Radiology, Memorial Sloan Kettering Cancer Centre, 10065 New York; USA.*
[d] *Institute of Organic Chemistry, University of Innsbruck, Innrain 80-82, Innsbruck, Austria.*

Contents:

1. General S2

2. Photoacoustic imaging S2

    Calculation of relative PA intensity S2

3. XTT-cell viability assay S3

4. *In vivo* multi-spectral optoacoustic imaging (MSOT) S3

5. Metal insertions into black metal-free porphyrins S4

    A) Synthesis of black Co$^{(II)}$ Porphyrin (**3-Co**)

    B) Synthesis of black Cu$^{(II)}$ Porphyrin (**3-Cu**)

    C) Tetranaphthoquinono Porphyrins **3-Zn**, **3-Ni**, **3-2H**

6. UV/Vis spectra S7

7. Fluorescence Emission of **3-Zn** S8

8. Phantom design images S9

9. PA spectroscopy S10

10. Stability of **3-Zn** in 10% fetal calf serum (FCS) S14

11. Stability of **3-Zn** to excess of free thiol (N-Ac-Cys-OMe) S16

12. MS spectra S18

13. References S19

1. General.

All the reactions, work-up and chromatography were performed under protection from light (wrapping in alumina foil). All the glassware for the reactions was oven dried at 100 °C and cooled under high vacuum (HV) before use and kept under argon. Absolute solvents were prepared by distillation over calcium hydride. Organic solutions were concentrated under reduced pressure on a Büchi rotary evaporator (< 37°C water bath). Thin-layer chromatography was carried out using Merck Kieselgel 60 F254 (230-400 mesh, 25x25cm for preparative scale). Solvents - dichloromethane (CH_2Cl_2), ethylacetate (EtOAc), and hexane were distilled before use from technical grade solvents. Methanol from Acros HPLC grade, $Cu(OAc)_2.H_2O$ (> 99% purity) from Merck, and anhydrous $CoBr_2$ from Acros (>99% purity) are used as received. UV/Vis spectra: *PerkinElmer Lambda 35 spectrometer*, in 10mm quartz cells, λ_{max} (log ε) in nm. Fluorescence spectra: *PerkinElmer LS45* or *Varian Cary Eclipse*. M**S**: MALDI (DHB matrix, Bruker Ultraflex MALDI TOF (Bruker Daltonic GmbH), *m/z* (rel. intensity %).

2. Photoacoustic Imaging: The pre-clinical PA device VEVO LAZR (from Visualsonics Inc, Amsterdam, NL) equipped with a LZ 250 transducer having a centre frequency of 21 MHz was used for scanning (26 db gain). The VEVO LAZR built-in pulse laser (wavelengths between 680-970 nm) was warmed up for 30 min before use and energy was calibrated using an internal sensor. Tube phantom experiments were carried at 9-11 mm depth in a water chamber with total of 59 (for 5 nm step) or 146 frames (2 nm steps). The Vevo 2100 software was used for data processing. During the measurements of serial dilutions, all variable parameters were kept constant i.e. photoacoustic gain, laser power, focus depth, frame averaging, and frame rate.

Calculation of relative photoacoustic intensity:

PA intensity (a.u) was measured in tube phantoms for all the solutions using identical regions of interest. The PA Avg signal/conc ratios of the black porphyrins were divided by the according ratio of ICG (then, for ICG relative PA intensity becomes 1).

3. Cell Viability XTT assay

In a 96-well plate, A549 cells ($5x10^3$ to $10x10^3$) were seeded and treated with DMEM media supplemented with 10% FCS, 1% pencillin/streptomycin (P/S). The cells were incubated at 37 °C (in 5% CO_2 incubator) for 24 h to let the cells attach to the surface of the 96-well plate. The substance in DMEM media (conc. 0.001 µmol/ml to 0.1 µmol/ml) was added to the above well plate, and further incubated at 37 °C in 5% CO_2 incubator for 24 h. XTT-solution was freshly prepared by XTT test kit (using the prescribed protocol from Gibco), and 50 µL of this mixture was added to each well of the above cells. After 2-4 h, the absorption was measured in TECAN-reader at 475 nm wavelength using 660 nm as reference.

4. *In vivo* multi-spectral optoacoustic imaging (MSOT)

Two dimensional (2D) static multispectral optoacoustic (MSOT) imaging was performed on a previously described inVision 256-TF system from iThera Medical (Munich, Germany) [S1, S2]. Female nude mice (4-6 weeks, n = 6, performed according to the Guidelines for the Care and Use of Animals for Research, approved by MSKCC's Institutional Animal Care and Use Committee) were intravenously injected with **3-Zn** (150 µL, 100 µM, 30% PEG300 in PBS, n=3) and PBS (150 µL, n=3). At 1 h post injection, mice were euthanized by asphyxiation with CO_2. Blood was collected via cardiac puncture and selected organs, liver, kidney, and muscle, were harvested for *ex vivo* MSOT imaging. Wavelengths from 700 nm to 900 nm in 20 nm steps (power: 100 mW) were used for excitation, with 5 acquisitions averaged per wavelength per frame. After image reconstruction, linear spectral unmixing was conducted to detect and select the specific signal of 'black' porphyrin from other intrinsic signal such as oxygenated and deoxygenated hemoglobin. All images were scaled to the same threshold (arbitrary units) to compare tissue injected with black porphyrin from PBS. To quantify the signal, the black porphyrin channel (green) was averaged over a circular area at the slices central to each tissue.

5. Metal insertions into black metal-free porphyrins

A) ***Tetranaphthoquinono Copper$^{(II)}$ Porphyrin* (3-Cu)**: In dry 10 mL round bottomed flask, 1.8 mg of (1.14 μmol) **3-2H** was taken under argon, 2.5 mL of CH_2Cl_2, 0.25 mL of MeOH (10:1, v/v) were added, followed by 4.6 mg of $Cu(OAc)_2.H_2O$ (23 μmol, 20 equiv.). The suspension was heated to reflux in a pre-heated oil-bath (at 50°C) under argon protecting from light. Reaction progress was followed through UV/Vis and TLC, after 1.5h the flask was removed from oil bath to cool down the reaction to room temperature. The resulting dark suspension washed with sat aq. $NaHCO_3$ (3 x 15 mL) and product mixture extracted into CH_2Cl_2 (*ca.* 4 x 10 mL) until the organic extracts were colourless. The combined organic extracts were filtered through a plug of dried cotton, and solvents removed under reduced pressure. The residue precipitated in MeOH/H_2O and dried under H.V. at 50°C for 15 h to obtain 1.7mg (1.03 μmol, 91%) of **3-Cu**.

UV/Vis (in CH_2Cl_2, c = 4.35 μM): λ_{max} (log ε): 724 (4.82), 661 sh (4.19), 545 (4.85), 413 (4.50), 365 (4.45), 253 (4.87).

^{1}H NMR: Paramagnetic compound.

MALDI-MS (DHB matrix, $C_{108}H_{100}N_4O_8Cu$; exact mass = 1643.68); base peak found at *m/z* (%):1624.10 (54), 1625.08 (80), 1626.06 (91), 1627. 04 (97), 1628. 01 (100), 1628.98 (90), 1629.94 (80), 1630.89 (80), 1631.86 (51), 1632.83 (28), 1633.79 (15), 1634.77 (7).

B) ***Tetranaphthoquinono Cobalt$^{(II)}$ Porphyrin* (3-Co)**: In an dry 10 mL round bottomed flask, 2 mg (1.26 μmol) of **3-2H** was taken, 2 mL of abs THF and 50 μlt of NEt_3 were added, followed by 5.5 mg of anhydrous $CoBr_2$ (25.1 μmol, 20 equiv.), and reaction was heated at 60°C for 3h. Resulting mixture washed with acidic water (pH 2, 3 x 15 mL) and product extracted into CH_2Cl_2 (*ca.* 4 x 10 mL), combined organic extracts were filtered through a plug of dried cotton, and solvents removed under reduced pressure. The residue precipitated in MeOH/H_2O to obtain pure product, which was dried under H.V. at 50°C for 15 h to obtain 1.9mg (1.1 μmol, 87%) of **3-Co**.

UV/Vis (in CH_2Cl_2, c = 3.67 μM): λ_{max} (log ε): 730 (4.67), 661 sh (4.14), 566 (4.65), 418 (4.43), 367 (4.44), 246 (4.90).

^{1}H NMR: Paramagnetic compound.

MALDI-MS (DHB matrix, $C_{108}H_{100}N_4O_8Co$; exact mass = 1639.69); Base peak found at *m/z* (%):1618.46 (54), 1619.43 (80), 1620.40 (91), 1621.39 (96), 1622. 04 (97), 1623.39 (100), 1624.29 (95), 1625.33 (84), 1626.22 (74), 1627.18 (70), 1628.15 (67), 1629.11 (58), 1630.09 (55), 1631.06(40), 1632.03 (36).

C) *Tetranaphthoquinono Porphyrins* 3-Zn, 3-Ni, 3-2H: Synthesis and analytical data were reported in [S3, S4].

<u>3-2H:</u> UV/Vis (in CH_2Cl_2, c = 38 μM): λ_{max} (log ε): 757 sh (4.33), 707 (4.72), 650 sh (4.31), 554 (5.03), 415 (4.63), 364 (4.40). **^{1}HNMR** (300 MHz, $CDCl_3$): -0.48 (s, 2H), 1.54 (s, 72H), 6.97 (s, 8H), 7.90-8.06 (br s, 8H), 8.18 (d, *J* = 1.6 Hz, 8H), 8.28 (t, *J* = 1.6 Hz, 4H). **MS** (**FAB**) $C_{108}H_{102}N_4O_8$: $(m/z)_{calcd.}$ = 1582.769 *m/z* (%): 1590.7 (14), 1589.6 (21), 1588.6 (31), 1587.6 (50), 1586.6 (76), 1585.6 (99), 1584.6 (100), 1583.6 (73, $[M+H]^+$), 1582.6 (61, M^+), 1581.6 (16), 1580.5 (8).

MALDI-MS (DHB matrix, $C_{108}H_{102}N_4O_8$; exact mass = 1582.769); base peak found at *m/z* (%):1572.68 (17), 1571.71 (44), 1570.74 (76), 1569.77 (100), 1568.79(92), 1567.82 (86), 1566.83 (56), 1565.84 (46), 1564.85 (28), 1563.87(19).

<u>3-Ni:</u> UV/Vis (in CH_2Cl_2, c = 10.4 μM): λ_{max} (log ε): 722.5 (4.89), 662 sh (4.22), 533.5 (4.87), 458 sh (4.35), 396 (4.49), 369 (4.47), 302.5 (4.55), 245 (4.89). **^{1}H NMR** (300 MHz, $CDCl_3$): 1.44 (s, 72H), 6.94 (s, 8H), 7.77 (d, *J* = 1.7 Hz, 8H), 7.83 (s, 8H), 8.15 (t, *J* = 1.7 Hz, 4H). **MS (FAB)** $C_{108}H_{100}N_4O_8Ni$: $(m/z)_{calcd.}$ = 1638.689, *m/z* (%): 1646.4 (25), 1645.4 (26), 1644.4 (39),

1643.5 (52), 1642.5 (87), 1641.5 (87), 1640.5 (94), 1639.5 (100, $[M+H]^+$), 1638.5 (25, M^+), 1637.4 (23).

<u>3-Zn:</u> UV/Vis (CH_2Cl_2, c = 9.71 μM): λ_{max} (log ε): 725 (4.90), 668 sh (4.29), 555 (4.95), 453.5 sh (4.52), 418 (4.66), 365 (4.53), 325 (4.39), 254 (4.99). ^1H NMR (300 MHz, in $CDCl_3$): 1.54 (s, 72H), 6.97 (s, 8H), 7.97 (s, 8H), 8.11 (br s, 8H), 8.33 (br s, 4H). FAB MS ($C_{108}H_{100}N_4O_8Zn$; exact mass = 1644.683). *m/z* (%): 1651.8 (32), 1650.8 (48), 1649.8 (71), 1648.8 (86), 1647.8 (100) 1646.8 (95), 1645.8 (79, $[M+H]^+$), 1644.8 (61, M^+).

X-ray structure analysis of 3-Zn: See reference [S3] (CCDC No. 699277)

6. UV/Vis Spectra

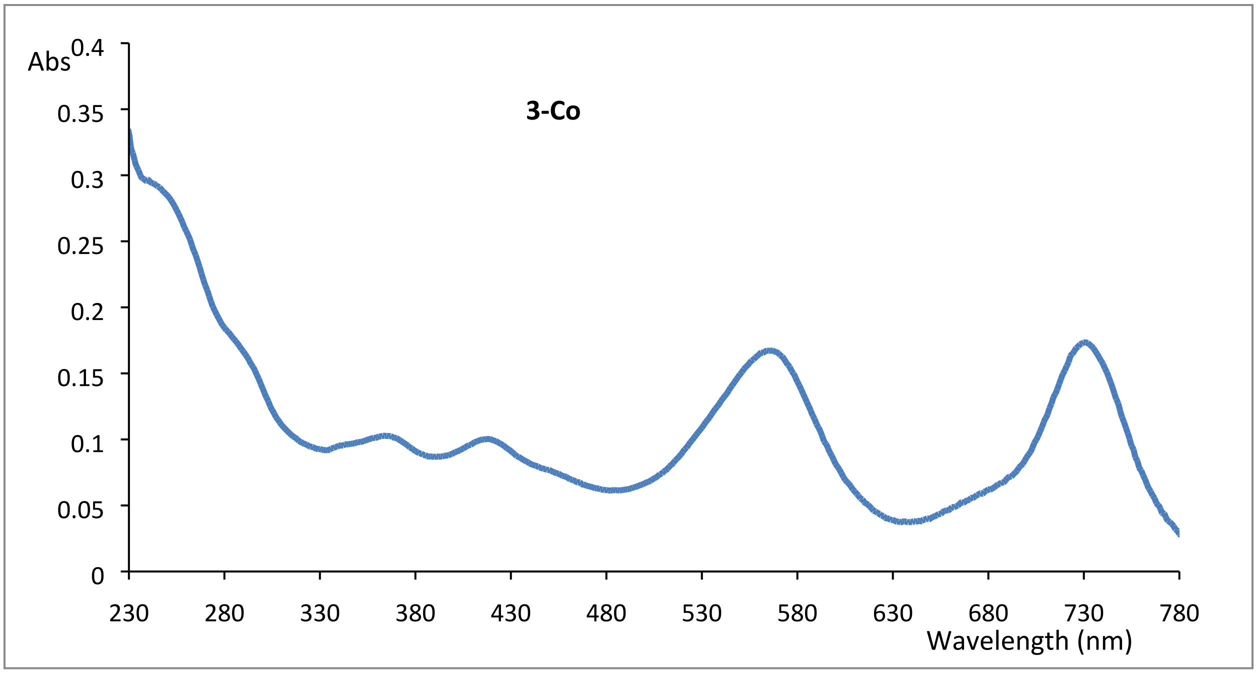

Fig. S1: UV/Vis-spectra of **3-Co** in CH_2Cl_2 (Conc. = 3.366 μM in CH_2Cl_2).

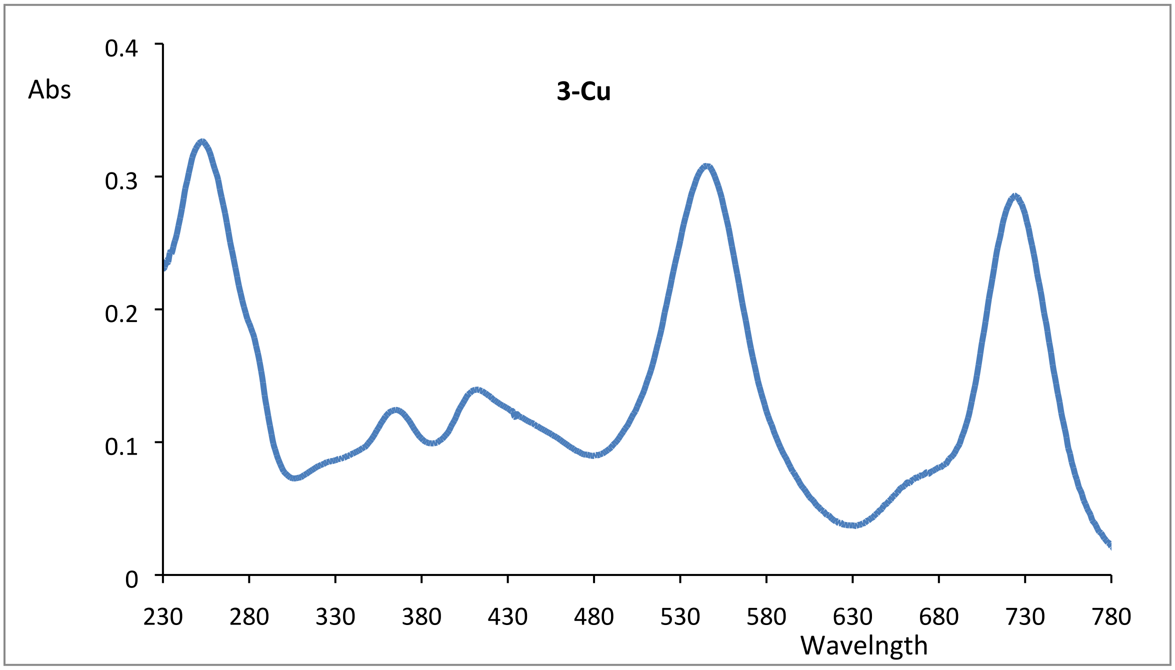

Fig. S2: UV/Vis-spectra of **3-Cu** in CH_2Cl_2 (Conc. = 4.356 μM in CH_2Cl_2).

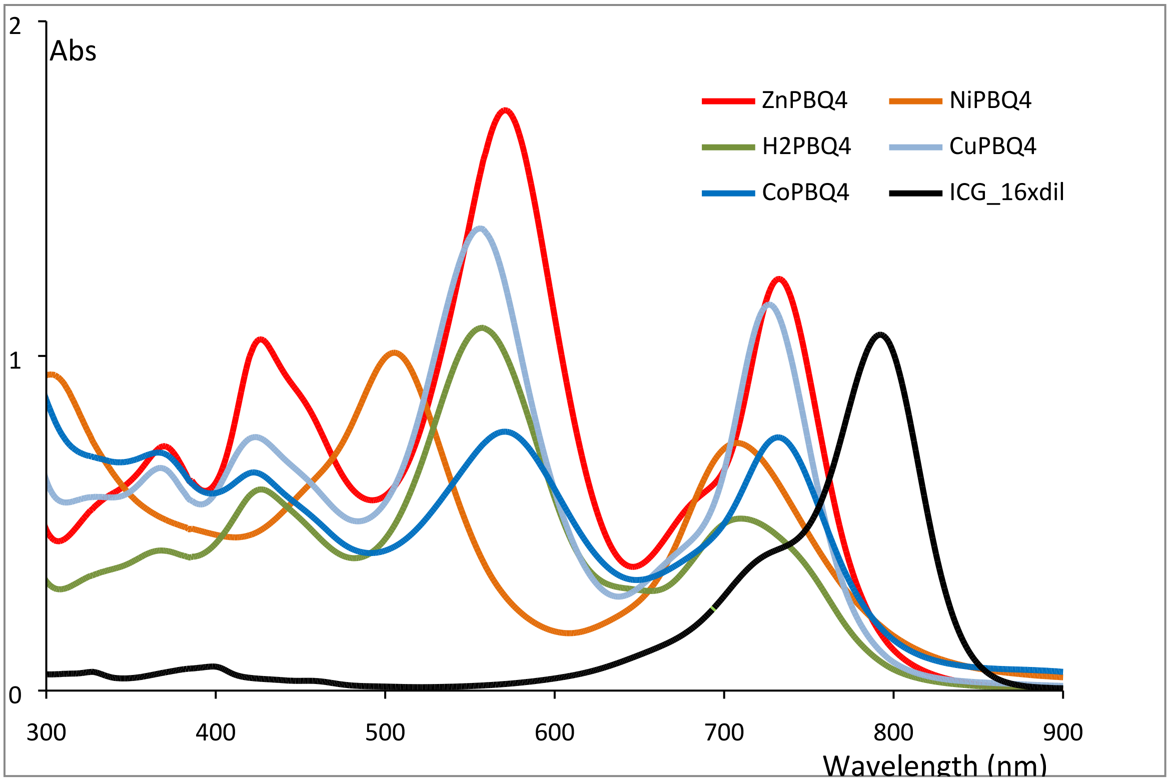

Fig S3: Overlay of UV/Vis-absorption spectra of naphthquinonoporphyrins (**3-M**) and ICG in DMF (concentrations: 16 µM of **3-2H** ($H_2P(BQ)_4$); 23 µM of **3-Zn**, ($ZnP(BQ)_4$); 26 µM of **3-Cu,** ($CuP(BQ)_4$); 31 µM of **3-Ni,** (NiP(BQ)4); 29 µM **3-Co** ($CoP(BQ)_4$); 11 µM of ICG).

7. Fluorescence spectra of 3-Zn

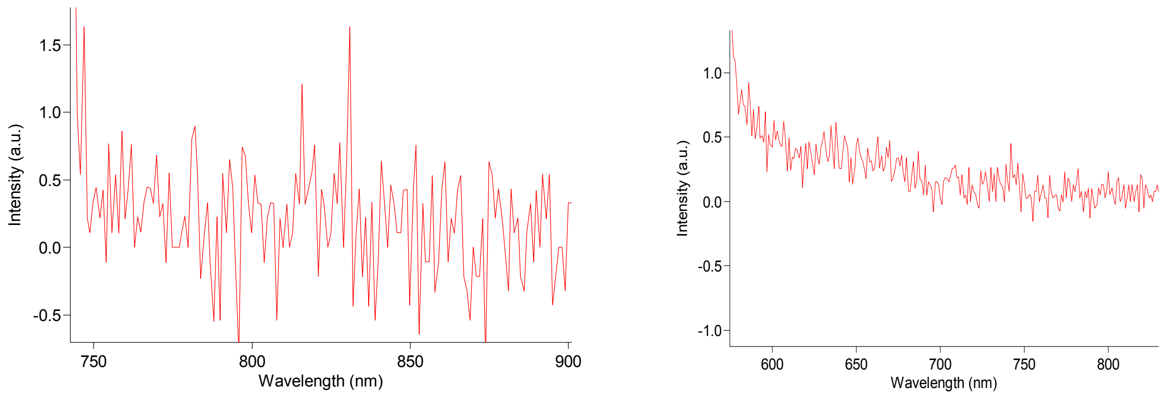

Fig S4: Fluorescence emission spectra of **3-Zn** (12 µM, 10% MeOH in CH_2Cl_2): Left at λ_{exc} = 736 nm and right at λ_{exc} = 567 nm.

8. Phantom design images

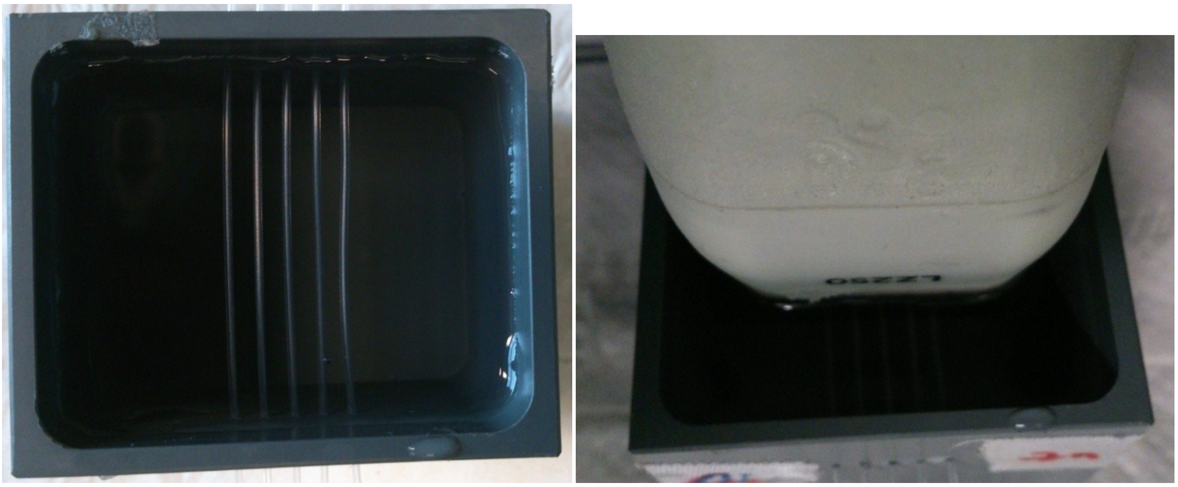

Fig S5: Images of the blood vessel mimicking water-chamber phantom used in this manuscript.

Chicken muscle phantom Imaging:

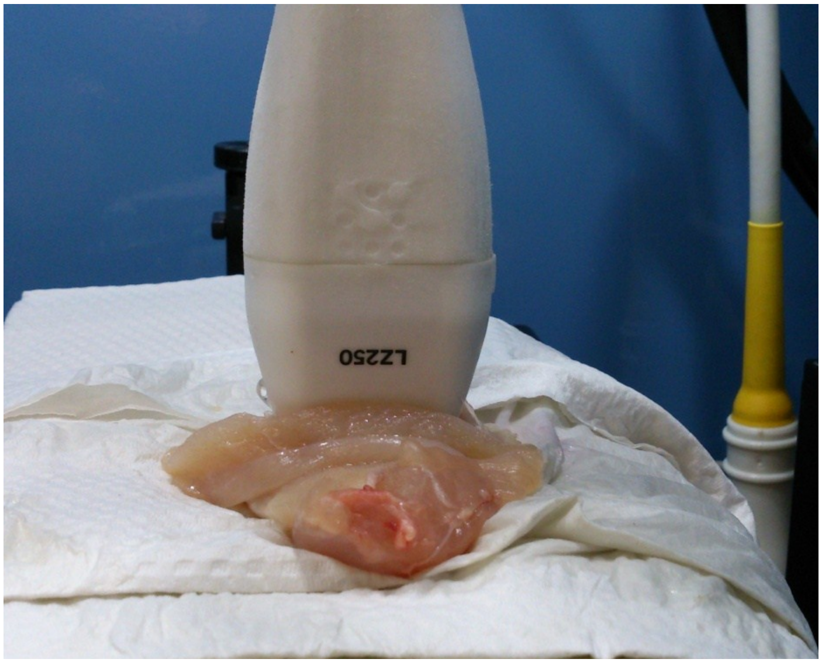

Fig S6: PAI of the chicken muscle phantom. A DMF solution containing the dyes was injected into the bottom layer of the chicken muscle, and thin layers of chicken muscle were placed on the top to generate an additional depth for the photoacoustic imaging.

9. PA spectroscopy

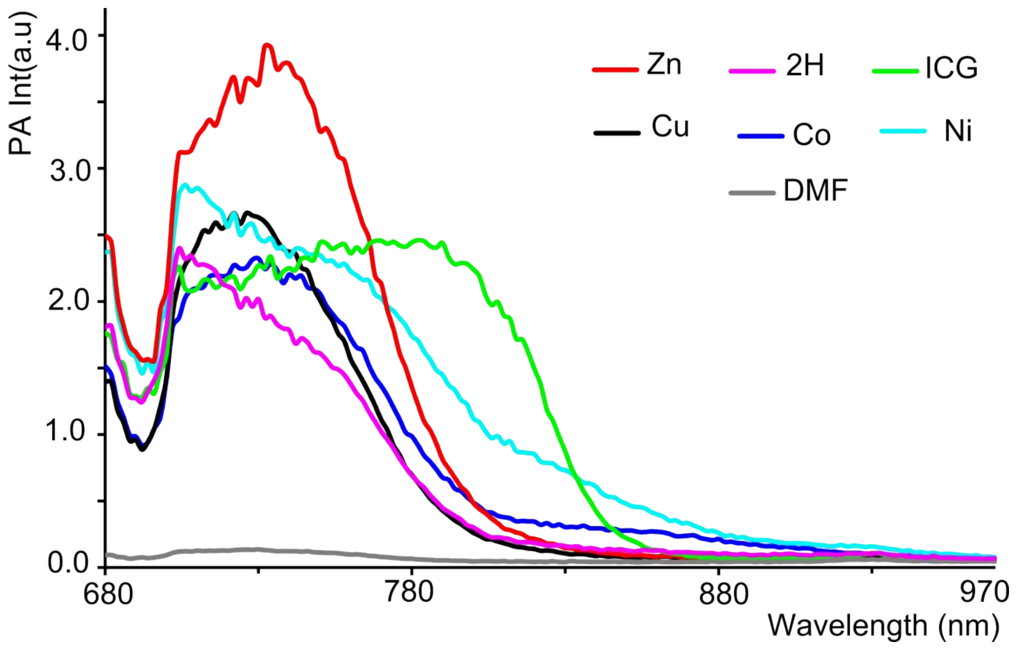

Fig S7: PA spectroscopy of the 'black' porphyrins **3-M** and ICG (in DMF, (concentrations: 63 µM of **3-2H**; 91 µM of **3-Zn**; 124 µM of **3-Ni**; 106 µM of **3-Cu**; 115 µM **3-Co**; 181 µM of ICG).

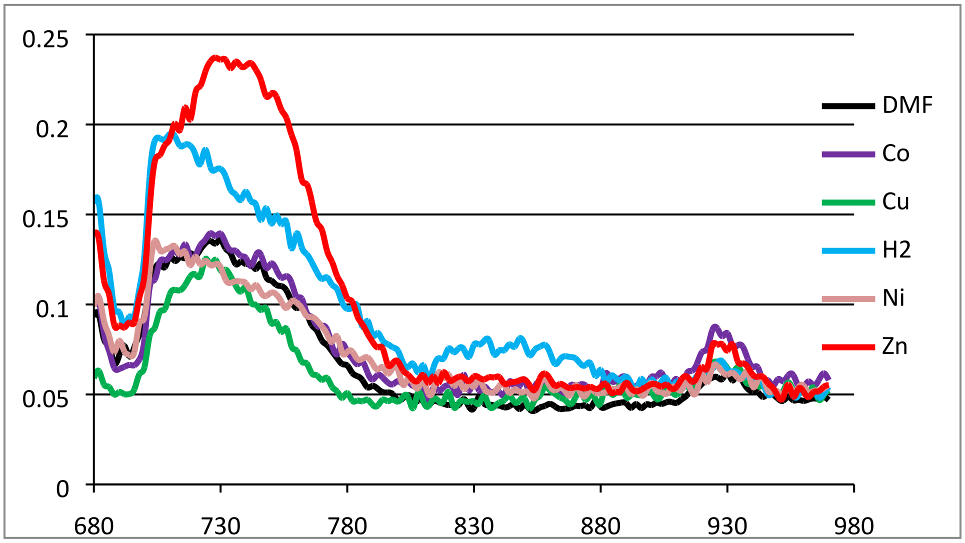

Fig S8: In DMF 20x diluted solutions of **3-M** PA spectroscopy (conc. = 3.2 µM of **3-2H**; 4.6 µM of **3-Zn**; 6.2 µM of **3-Ni**; 5.3 µM of **3-Cu**; 5.8 µM **3-Co**; 9 µM of ICG).

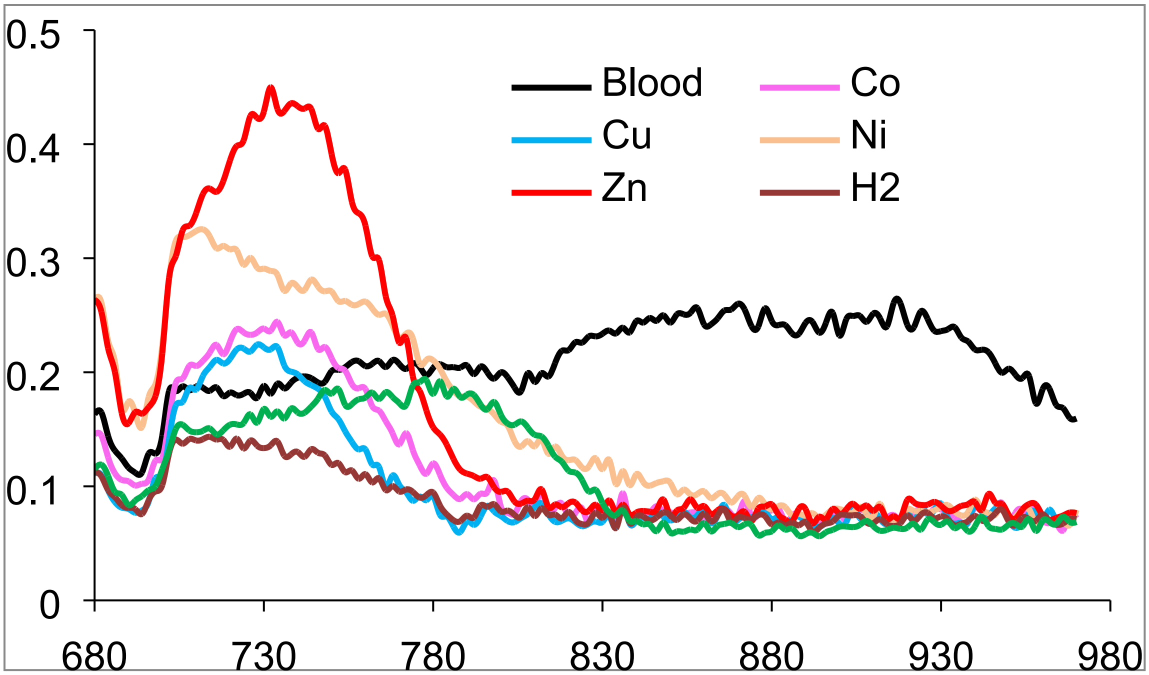

Fig S9: PA spectroscopy in 4x whole blood dilutions (conc. = 15.7 µM of **3-2H**; 22.8 µM of **3-Zn**; 31 µM of **3-Ni**; 26.5 µM of **3-Cu**; 28.8 µM **3-Co**; 45.2 µM of ICG).

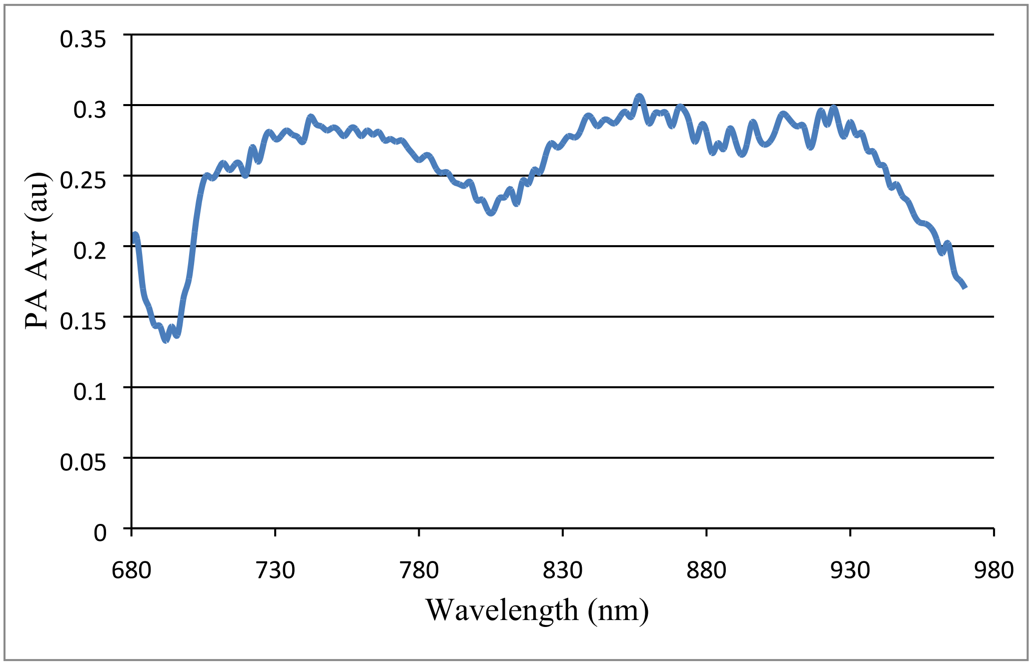

Fig S10: PA spectroscopy of whole blood (Swine, injected into a tube phantom).

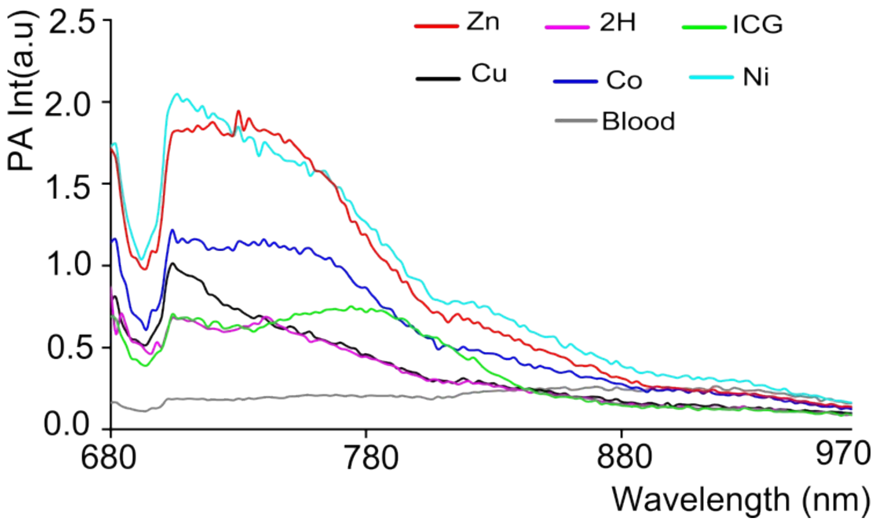

Fig S11: Blood PA Avr unsubtracted PA spectroscopy of **3-M**s in whole blood, Conc. in μM, **3-2H** (42), **3-Co** (76.6), **3-Cu** (70.6), **3-Ni** (82.6), **3-Zn** (60.6), and ICG (120.6).

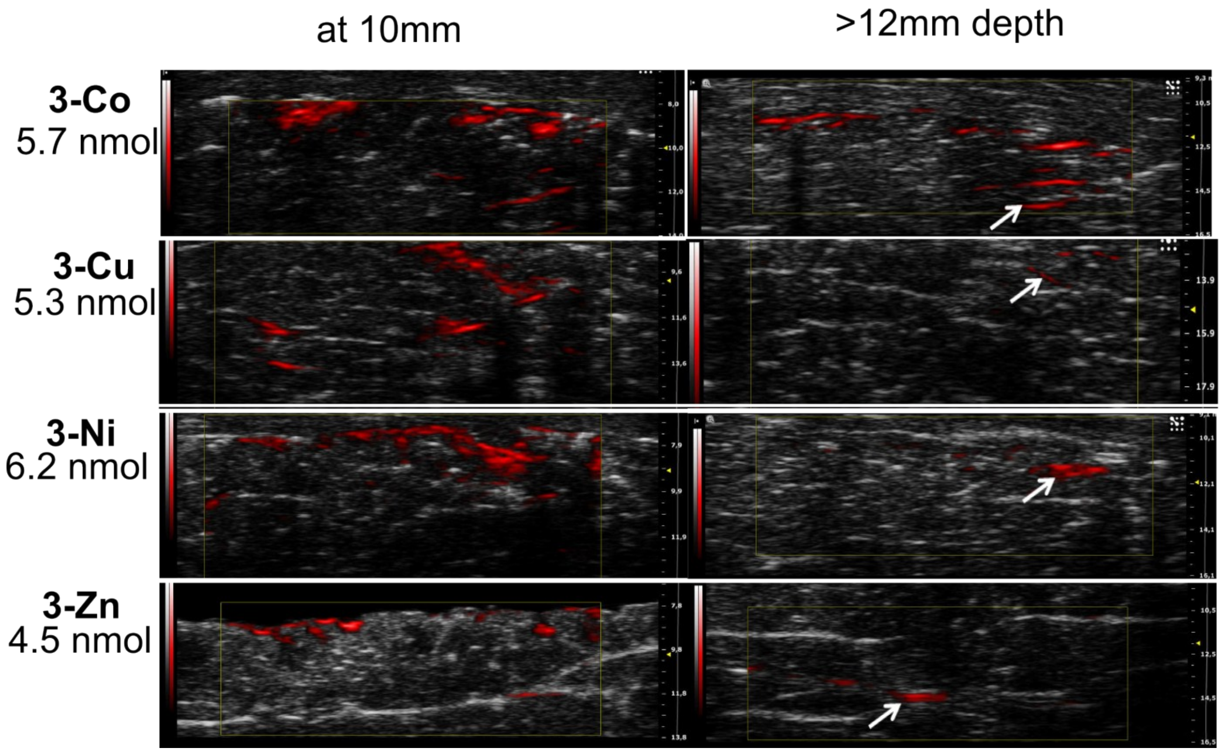

Fig S12: PA images of **3-M** in chicken muscle phantom (Ni: 720 nm, Cu: 726 nm, Co and Zn: 730nm).

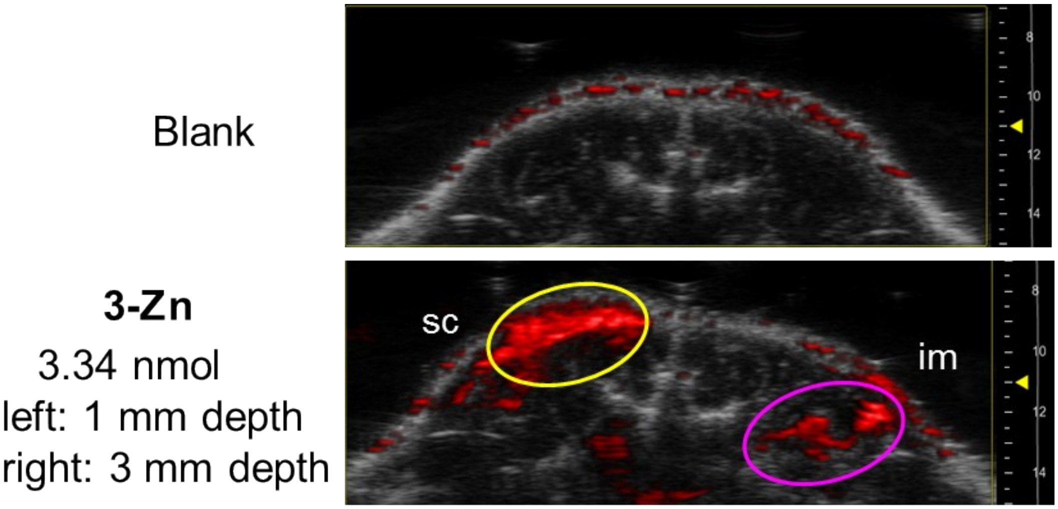

Fig S13: PA snapshot image (at 720nm) of **3-Zn** injected dead mouse at different depths (s.c = subcutaneous, 1 mm deeper, im = intramuscular, 3 mm deeper).

10. Stability of 3-Zn in 10% fetal calf serum (FCS) in PBS buffer

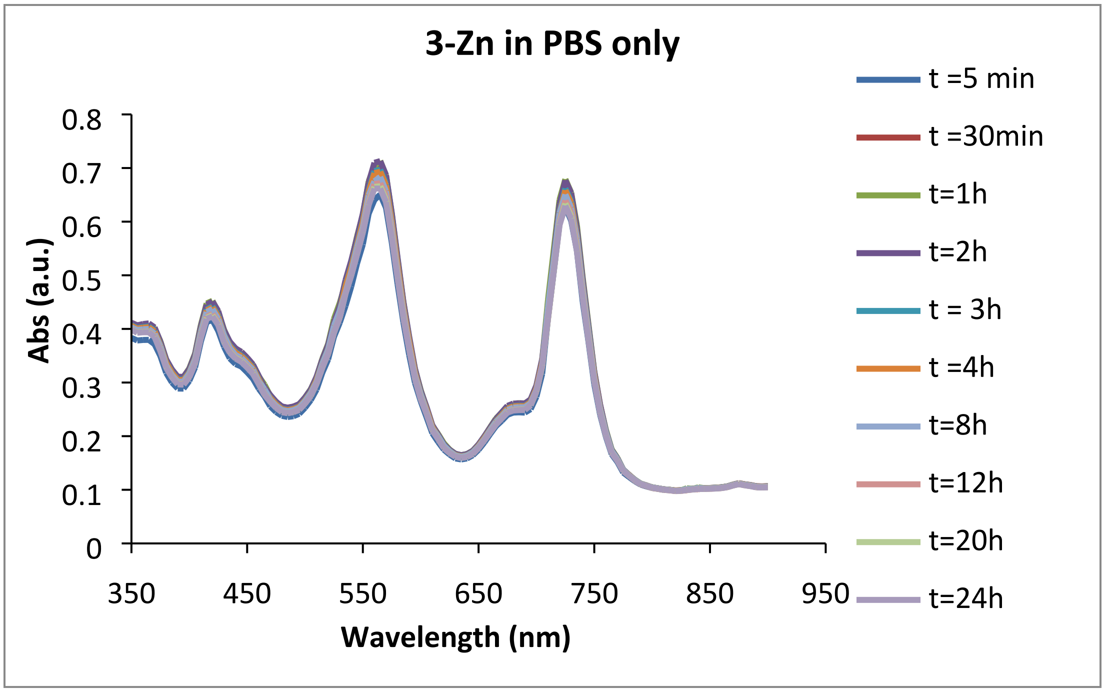

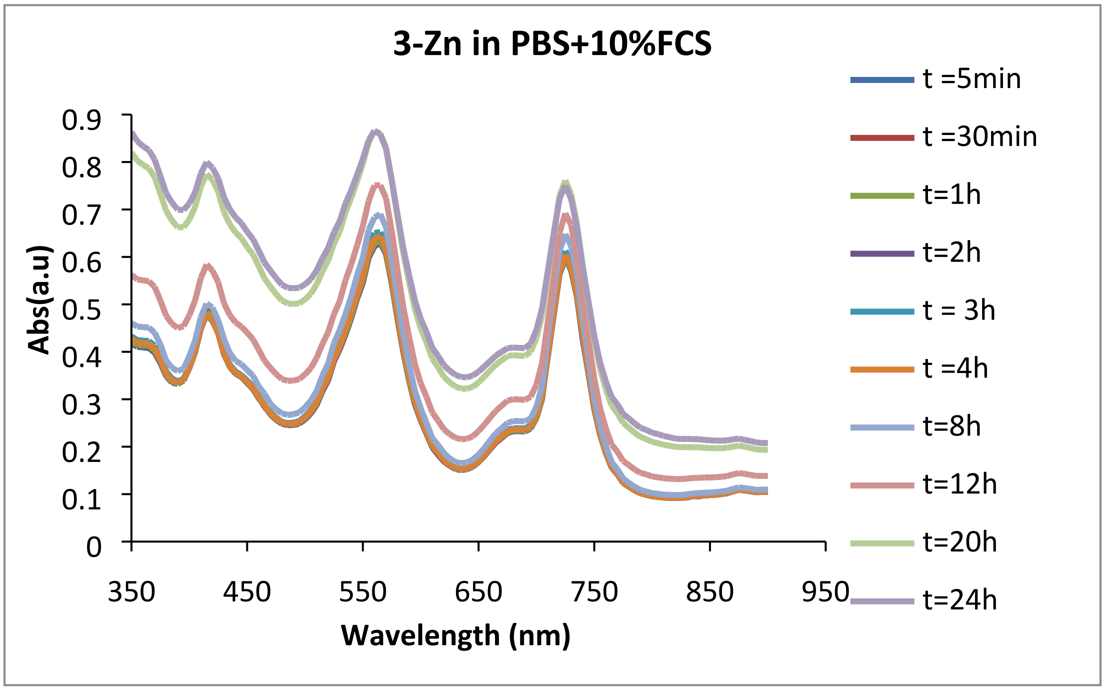

Fig S14: UV-Vis spectroscopy of **3-Zn:**cremophore EL in PBS (Top), and of **3-Zn:**cremophore in 10% FCS contained PBS (bottom) (average of 3 measurements; conc.: 15 µL of 1mM of **3-Zn** solution to 200 µL volume per well) (over 24h, some solution evaporation has caused a little shift in maxima of the 2nd spectrum).

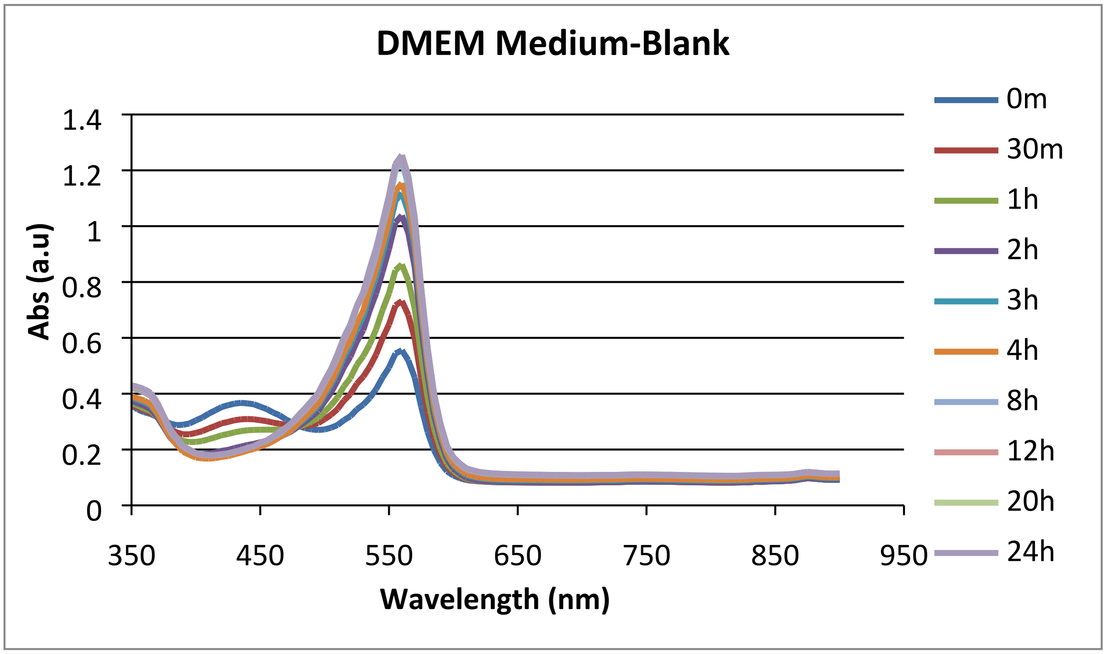

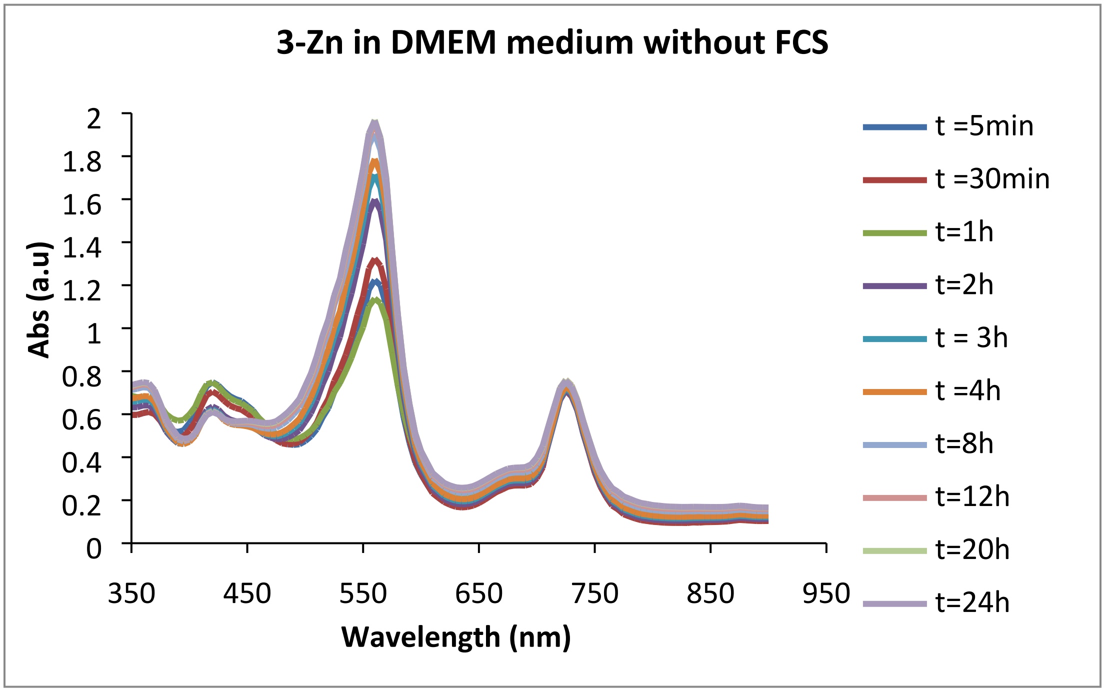

Fig S15: UV-Vis spectroscopy of **3-Zn:**cremophore in DMEM medium (Top), and of **3-Zn:**cremophore in 10%FCS contained DMEM medium (bottom) (average of 3 measurements, conc: 15 μL of 1mM of **3-Zn** solution volume in well: 200 μL; i.e. 75 nmol/ml).

11. Stability of 3-Zn to excess of free thiol (N-Ac-Cys-OMe): In a HPLC vial, 0.3 mg of **3-Zn** in ethylacetate (1 ml) was taken, added 2 mg of N-acetylated cysteine methylester (Ac-Cys-OMe, Sigma Aldrich), and reaction was followed by injecting 5 μL of the mixture at different time points in supercritical fluid chromatography (SFC, column: Kromasil-Si-60, method: 4ml_10min_35ml_gradient).

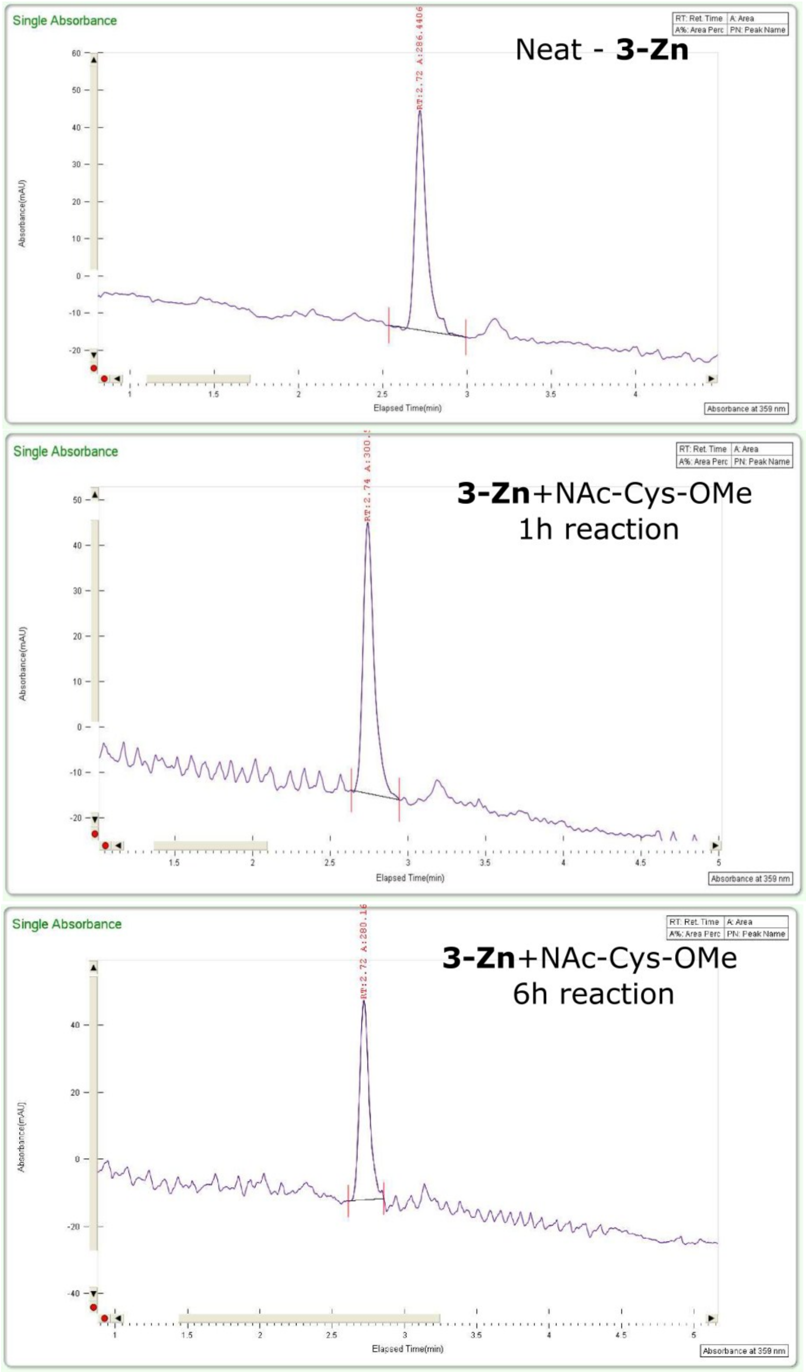

Fig S16: Supercritical fluid chromatography (SFC-) traces of **3-Zn** reaction with N-acetyl-cysteine-methylester (1h, 6h) (Similar retention times can be seen in each chromatogram).

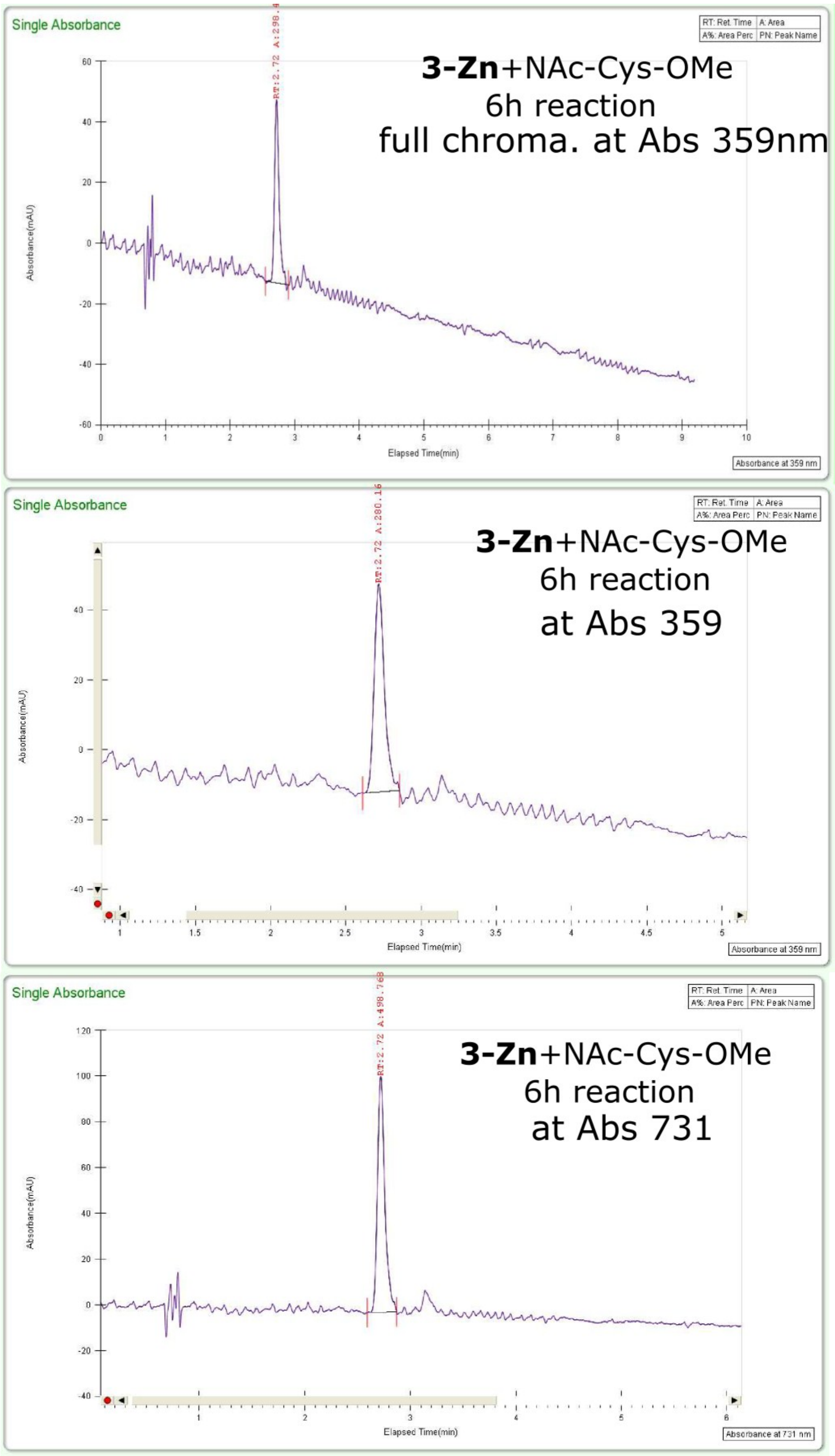

Fig S17: SFC-trace of **3-Zn** reaction with N-acetyl-cysteine-methylester after 6h (in 10 min elution at Absorbance at 359 and at 731 nm).

12. Mass spectra

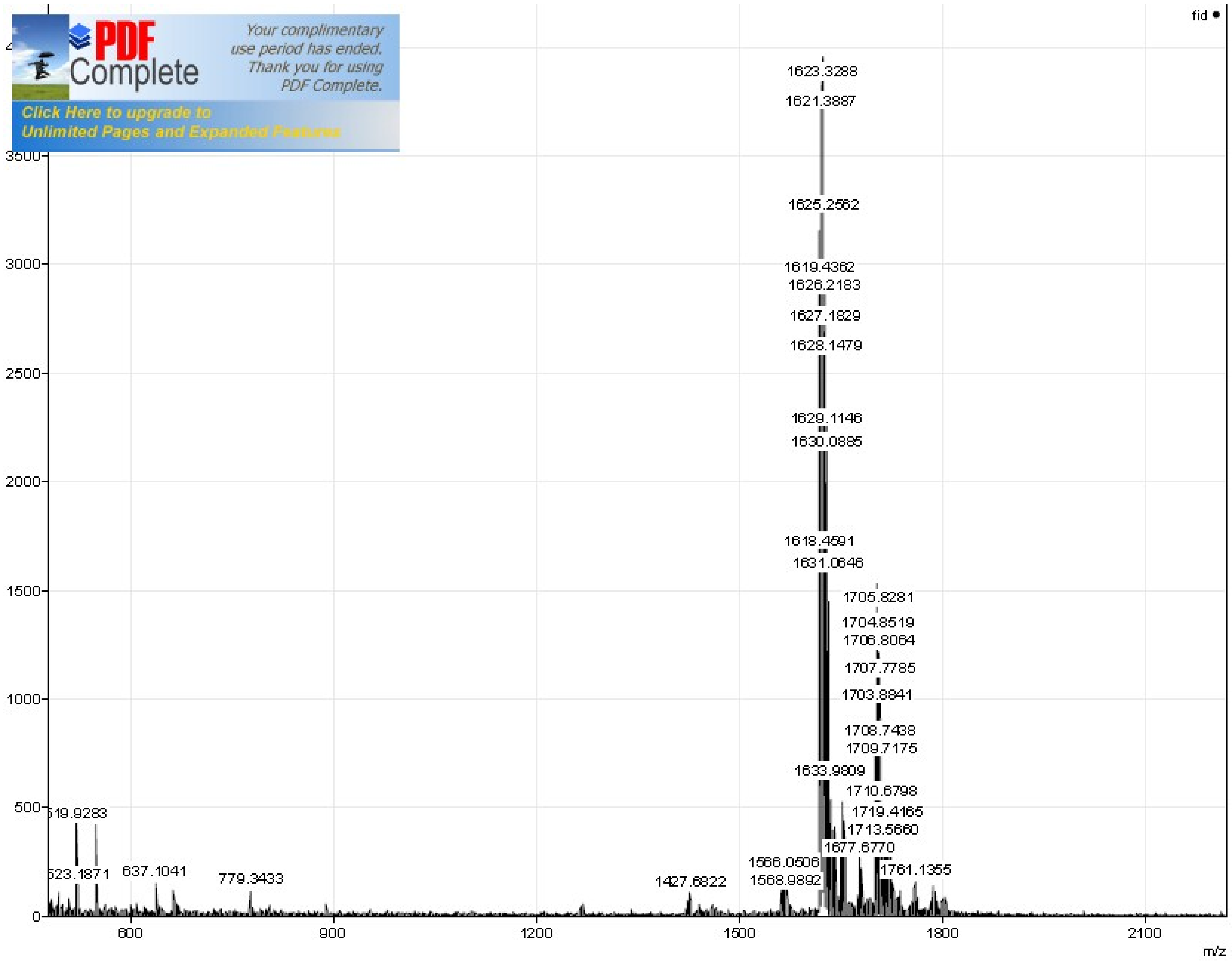

PDF Complete
Your complimentary use period has ended. Thank you for using PDF Complete.
Click Here to upgrade to Unlimited Pages and Expanded Features

Fig S18: MALDI (in DHB) spectra of **3-Co**.

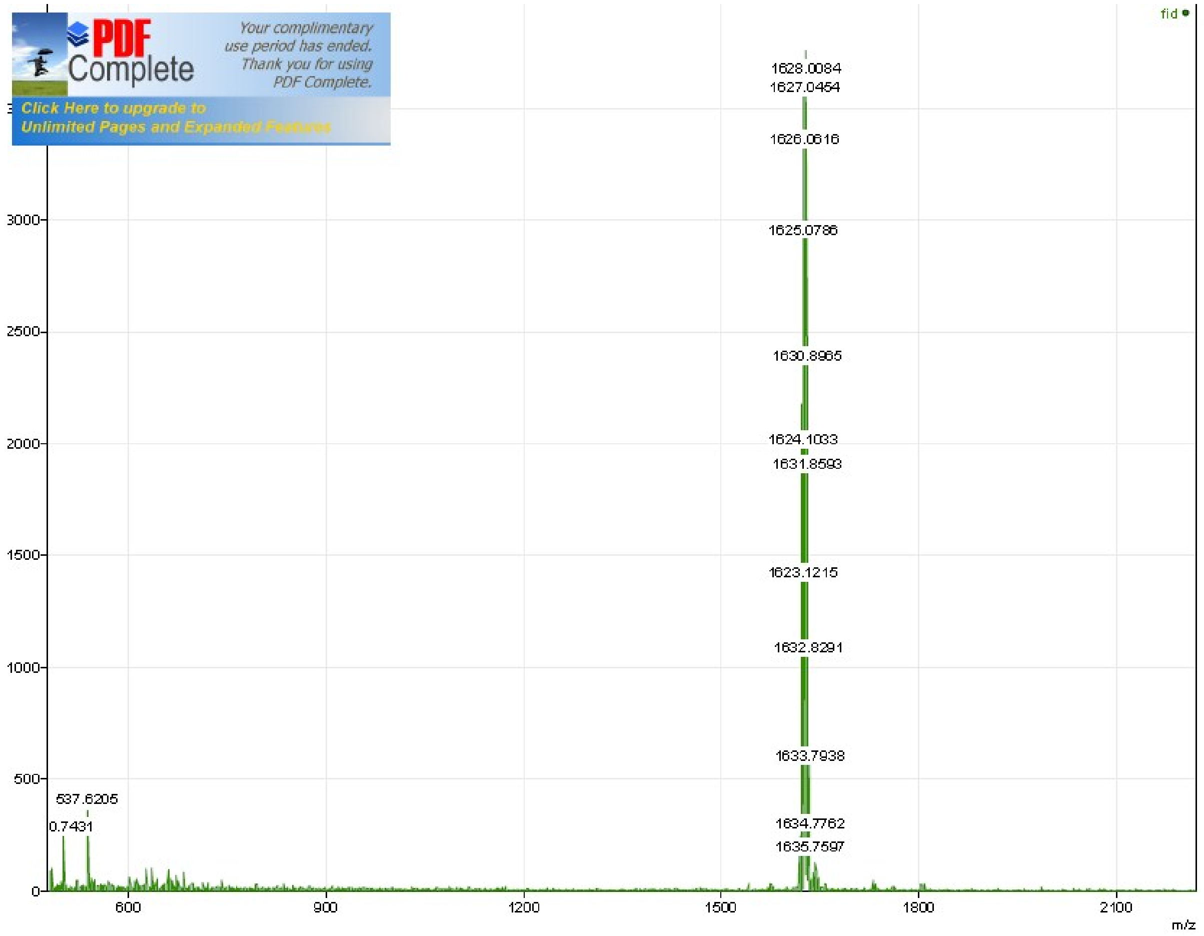

PDF Complete
Your complimentary use period has ended. Thank you for using PDF Complete.
Click Here to upgrade to Unlimited Pages and Expanded Features

Fig S19: MALDI (in DHB) of **3-Cu**.

13. References:

S1. N.C. Burton, M. Patel, S. Morscher et al. *Neuroimage* **2013**; *65,* 522.

S2. V. Neuschmelting, H. Lockau, V. Ntziachristos, J. Grimm, *Radiology*. **2016**; *280*,137

S3. S. Banala, T. Rühl, P. Sintic, K. Wurst, B. Kräutler, *Angew. Chem. Int. Ed* **2009**, *48*, 599.

S4. S. Banala, K. Wurst, B. Kräutler*, ChemPlusChem* **2016**, *81*, 477.
